# Applying expertise: Extrapleural pneumonectomy and extended pleurectomy decortication in the management of Masaoka-Koga stage IV thymoma

**DOI:** 10.1016/j.xjon.2025.04.010

**Published:** 2025-04-24

**Authors:** Karishma Chandarana, Marinos Koulouroudias, Helen Weaver, Apostolos Nakas

**Affiliations:** Department of Thoracic Surgery, Glenfield Hospital, University Hospitals of Leicester Trust, Leicester, United Kingdom

**Keywords:** thymoma, mediastinum, extended pleurectomy decortication, EPD, extrapleural pnuemonectomy, EPP

## Abstract

**Objectives:**

The European Society of Medical Oncology supports the use of surgery with adjuvant radiotherapy in resectable Masaoka-Koga Stage IV thymomas. We explore the role of extended pleurectomy decortication (EPD) and extrapleural pneumonectomy (EPP) in the management of patients with Masaoka-Koga stage IV thymic tumors with pleural involvement from our single-center experience.

**Methods:**

We conducted a retrospective analysis of patients who had undergone extended resections over a 10-year period for Masaoka-Koga stage IV thymomas at our thoracic unit in the United Kingdom. Data was gathered from patient records and electronic databases.

**Results:**

Ten patients were included in our series; 90% with primary thymoma and 10% with metastatic recurrence. In total, 80% of patients had EPD and 20% EPP; 60% had pericardium resected and 50% the ipsilateral hemidiaphragm. Length of stay was 2 to 21 days (median, 7 days). There was no in-hospital or 90-day mortality. Histology subtypes were variable: World Health Organization type AB (20%), B1 (10%), B2 (50%), and B3 (20%). A total of 60% of patients had R1 resection. All patients had adjuvant therapy. In total, 70% of patients had disease recurrence with an average disease-free interval of 44 months (range, 8 months to 10 years). Five-year survival was 90% with an overall survival of 60%.

**Conclusions:**

This series supports the use of extended resections in selected patients with Masaoka-Koga stage IV thymoma as part of multimodality treatment. EPP and EPD are not part of routine thoracic surgery practice in the United Kingdom. We suggest these cases are referred to dedicated centers with required expertise.

**Figure:**
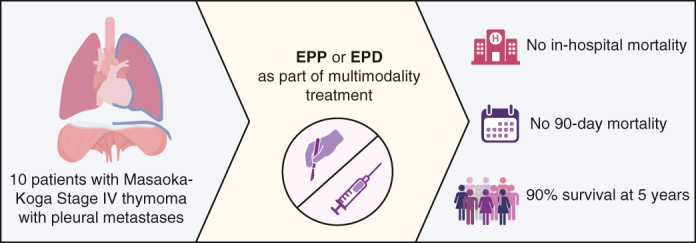
Applying expertise: EPP and EPD for Masaoka-Koga stage IV thymoma with pleural metastases.

**Central Message**

Extended pleurectomy decortication and extrapleural pneumonectomy may have a role in the treatment of Masaoka-Koga stage IV thymoma and can be recommended as part of multimodality treatment.

**Perspective**

This article provides surgical perspectives and a creative approach to Masaoka-Koga stage IV thymoma with use of extended pleurectomy decortication (EPD) and extrapleural pneumonectomy (EPP), classically used for mesothelioma. Modern thoracic oncology relies on multimodality treatment and multidisciplinary expertise; here, we challenge the limits of unresectable disease to improve patient survival.

Thymic epithelial tumors are the most common tumor of the anterior mediastinum.^1^^,^^2^ They vary in size, anatomy, and pathologic type and can range from slow-growing, indolent tumors to highly aggressive ones. Thymic epithelial tumors with local invasion remain a relatively rare and challenging clinical entity. Multimodality treatment aims to maximize survival by preventing recurrence.^1^^,^^3^

Current European Society of Medical Oncology (ESMO) guidelines support the use of surgery with adjuvant radiotherapy in Masaoka-Koga stage IV thymoma and thymic carcinoma in cases in which the tumor is considered to be resectable. Complete resection is the single most important factor when predicting recurrence.^4^ Similarly, ESMO guidelines recommend surgery for recurrent thymoma if complete resection is achievable. There is no standardized approach for the type of surgery recommended in the literature,^5^ and it is therefore largely dependent on the disease distribution and the knowledge, expertise, and resources available to the multidisciplinary team (MDT). The European Society of Thoracic Surgeons conducted a multicenter retrospective analysis of 152 patients, which found no statistical difference in disease-free survival, or overall survival, in patients with local or advanced disease undergoing extrapleural pneumonectomy (EPP), total pleurectomy, or local pleurectomy.^6^

Because surgery for thymic epithelial tumors with pleural involvement is infrequently performed, clinical trials and prospective studies are difficult to undertake; hence, the value of surgical therapy for primary or recurrent thymic tumors with pleural involvement is determined on the basis of retrospective observational data.

We report our experience of extended resections for patients with Masaoka-Koga stage IV thymic tumors with pleural involvement undergoing extended pleurectomy decortication (EPD) and EPP; procedures classically developed for malignant pleural mesothelioma (MPM) and tuberculosis surgery.^7^^,^^8^

## Methods

We retrospectively identified and reviewed patients at our thoracic unit who had undergone extended resections over a 10-year period (2012-2022) for Masaoka-Koga stage IV thymoma. Data was collected from patient records and electronic databases, including demographics, operative records, pathologic subsets, and postoperative outcomes. Sex was defined biologically as male or female, based on documentation in medical records.

The Governance committee of the University Hospitals of Leicester approved the study protocol and publication of data (registration number 14091, May 16, 2024). Data is available upon request. Patient written consent for the publication of the study data was waived. The recommendation for surgery was determined by the MDT, and patients received standard of care treatment.

A thymectomy was performed in addition to 1 of 2 extended resections: an EPD or an EPP. An EPD involves removal of all macroscopic disease with complete resection of the ipsilateral parietal and visceral pleura, as well as the diaphragm and pericardium, if there is disease in these areas. An EPP is defined as en bloc removal of ipsilateral lung, parietal pleura, pericardium, and diaphragm.^7^^,^^9^ In both procedures, the diaphragm and pericardium are reconstructed with prosthetic patches before closure.

## Results

In total, 98 patients who had thymectomy for thymoma over a 10-year period were identified from a pathologic database; however, only 10 of these patients underwent an EPD or EPP during this time. Ten patients were included in this case series, of whom 9 were female. The median age was 45 years (range, 35-63 years). Three patients had confirmed diagnosis of myasthenia gravis preoperatively (Table 1). Nine patients underwent surgery for primary thymoma and 1 for metastatic recurrence (Table 2).

**Table 1:** Patient demographics

Patient	Year	Sex	Age at operation, y	Myasthenia gravis?
1	2019	F	35	N
2	2015	F	40	N
3	2017	F	53	N
4	2017	F	48	Y
5	2014	F	49	N
6	2022	F	42	N
7	2019	F	36	N
8	2012	F	41	N
9	2021	M	59	Y
10	2019	F	63	Y

*F*, Female; *N*, no; *Y*, yes; *M*, male.

**Table 2:** Management summary

Patient	Primary/recurrent thymoma	Treatment prior to resection	Staged procedure	Operation	Adjuvant therapy
1	Primary	Chemotherapy	N	EPD	Radiotherapy
2	Primary	None	N	EPD	Radiotherapy
3	Primary	Chemotherapy	N	EPD	Radiotherapy
4	Primary	None	N	EPD	Radiotherapy
5	Primary	Chemotherapy	Y	EPD	Radiotherapy
6	Primary	Chemotherapy	N	EPP	Radiotherapy
7	Primary	Chemotherapy	N	EPP	Radiotherapy
8	Recurrent	None for this episode	N	EPD	Chemotherapy
9	Primary	Chemotherapy	N	EPD	Radiotherapy
10	Primary	Chemotherapy	Y	EPD	Chemotherapy

*N*, No; *EPD*, extended pleurectomy decortication; *Y*, yes; *EPP*, extrapleural pneumonectomy.

All patients were referred to, and discussed at, a local lung cancer MDT. In total, 7 of 10 patients had chemotherapy before surgical resection because the tumor was deemed “borderline resectable” or “unresectable” at this stage (Table 2). Chemotherapy was in the form of cyclophosphamide, doxorubicin, and cisplatin, with additional prednisolone in a few cases. Most patients received 6 cycles; 1 patient received only 4 cycles because of a lack of response. Five of 7 patients had partial response, and 2 of 7 had stable disease as per Response Evaluation Criteria in Solid Tumours (RECIST) criteria.^10^

All cases were rediscussed at the MDT before proceeding with resection. Preoperative workup included assessment of performance status, pulmonary function tests and an echocardiogram. Seven of 10 patients had computed tomography (CT)-positron emission tomography (PET) scans before resection to assess nodal disease or distant spread, with the remainder having at least CT imaging of the chest, abdomen, and pelvis.

**Figure E1:**
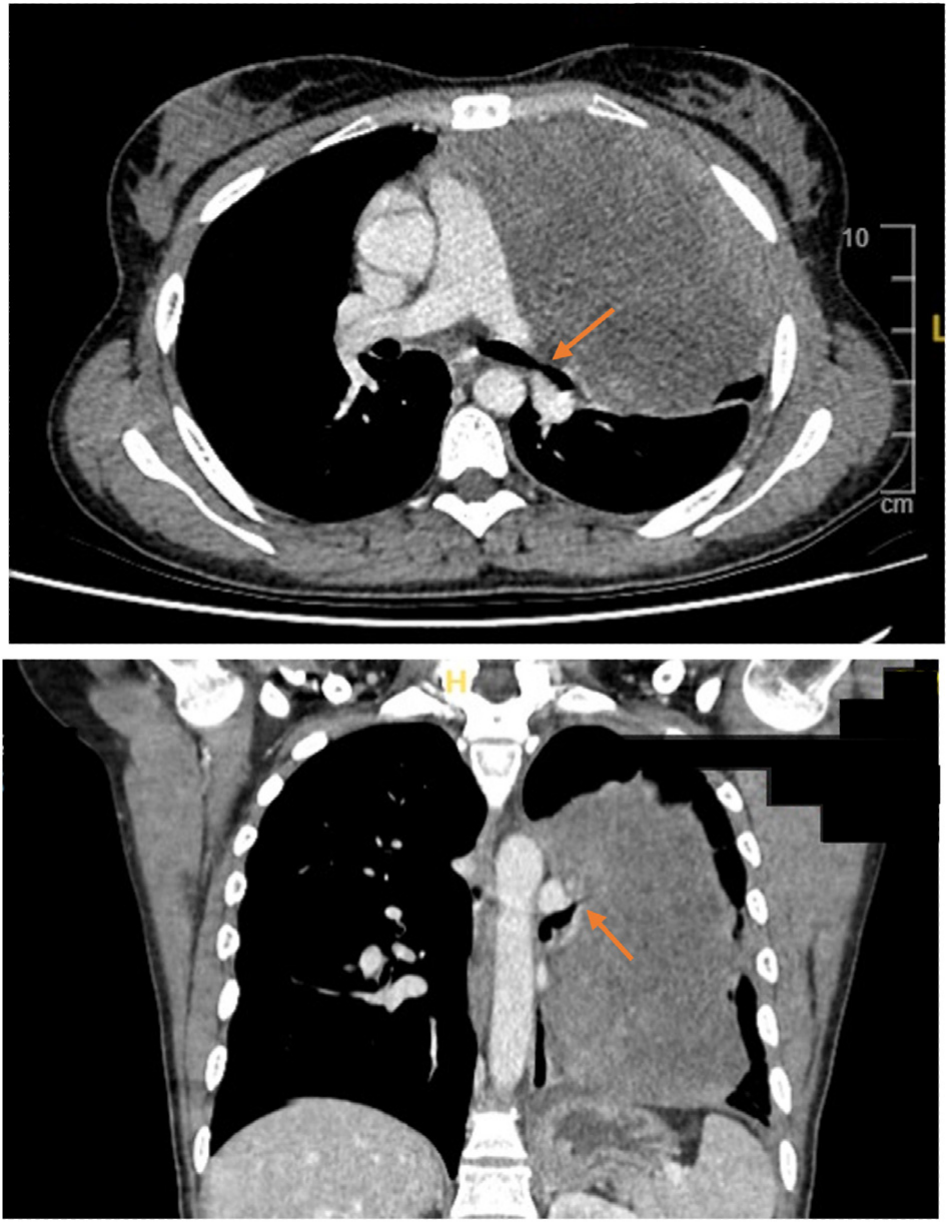
*Arrow* marks sizeable tumour and invasion of left main pulmonary artery necessitating left pneumontectomy. *Arrow* marks hilar invasion and sizeable tumour demonstrating proximity and invasion with left main pulmonary artery necessitating left pneumontectomy.

Each case posed individual challenges and required careful surgical planning. Eight patients had an EPD and 2 patients had an EPP (Table 3). The indication for EPP was tumor invasion into the hilum or extensive lung parenchymal involvement (Figures E1 and 1). In both procedures, a total parietal pleurectomy was performed on the ipsilateral side, in addition to a pneumonectomy in an EPP, or a total visceral pleurectomy in an EPD. The extent of resection was determined on the basis of underlying tumor biology and the assumption that micrometastatic disease would remain unresected. Care was taken to not breach the capsule of the thymoma avoiding seeding. The pleural cavity was washed with warm water after the majority of disease resection, but before resection of pericardium/diaphragm. Once the diaphragm was reconstructed, a water wash was performed again with the patient's head down in order to avoid crosscontamination into the abdomen. Reconstruction was with Johnson & Johnson's VICRYL (polyglactin 910) mesh for pericardium and GORE DUALMESH biomaterial for diaphragm.

**Table 3:** Operative details

Patient	Laterality	Staged procedure	Operation	Surgical approach	Diaphragm resected	Pericardium resected	Lung parenchyma resected	Laser assisted
1	Right	N	EPD	Sternotomy	N	N	Y	N
2	Left	N	EPD	Sternotomy + thoracotomy	N	Y	Y	N
3	Left	N	EPD	Sternotomy + thoracotomy	Y	N	Y	N
4	Right	N	EPD	Sternotomy	N	N	Y	N
5	Left	Y	EPD	Sternotomy + thoracotomy	N	Y	Y	N
6	Left	N	EPP	Sternotomy + thoracotomy	Y	Y	Y	N
7	Right	N	EPP	Thoracotomy	Y	Y	Y	N
8	Right	N	EPD	Thoracotomy	N	N	N	Y
9	Left	N	EPD	Thoracotomy	Y	Y	Y	Y
10	Right	Y	EPD	Sternotomy + thoracotomy	Y	Y	N	Y

*N*, No; *EPD*, extended pleurectomy decortication; *Y*, yes; *EPP*, extrapleural pneumonectomy.

**Figure 1:**
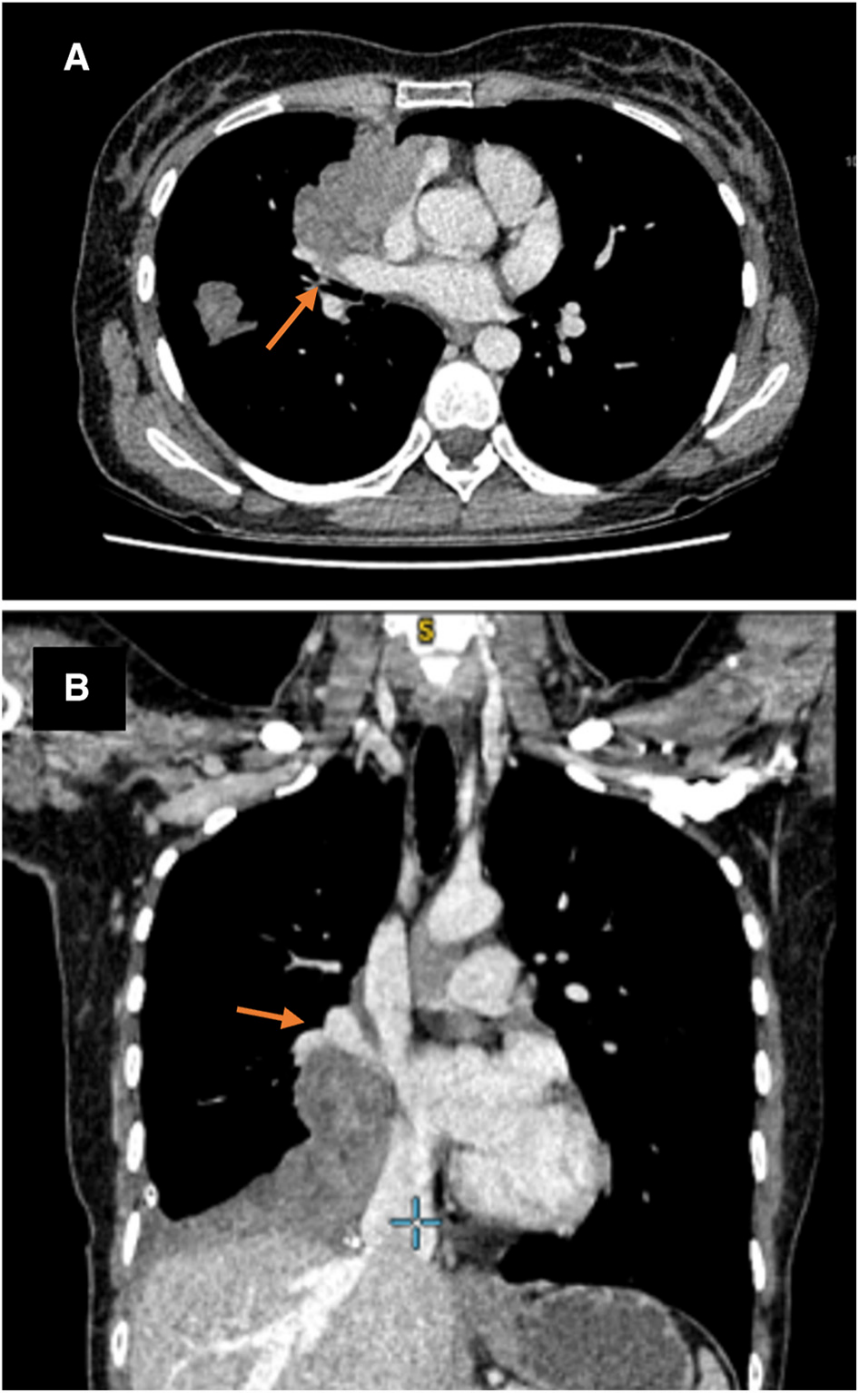
Axial computed tomography scan of the chest with contrast. Arrows show involvement of right main pulmonary artery at origin of upper lobe (A); and effacement of inferior pulmonary vein found to be involved intraoperatively (B).

**Figure 2:**
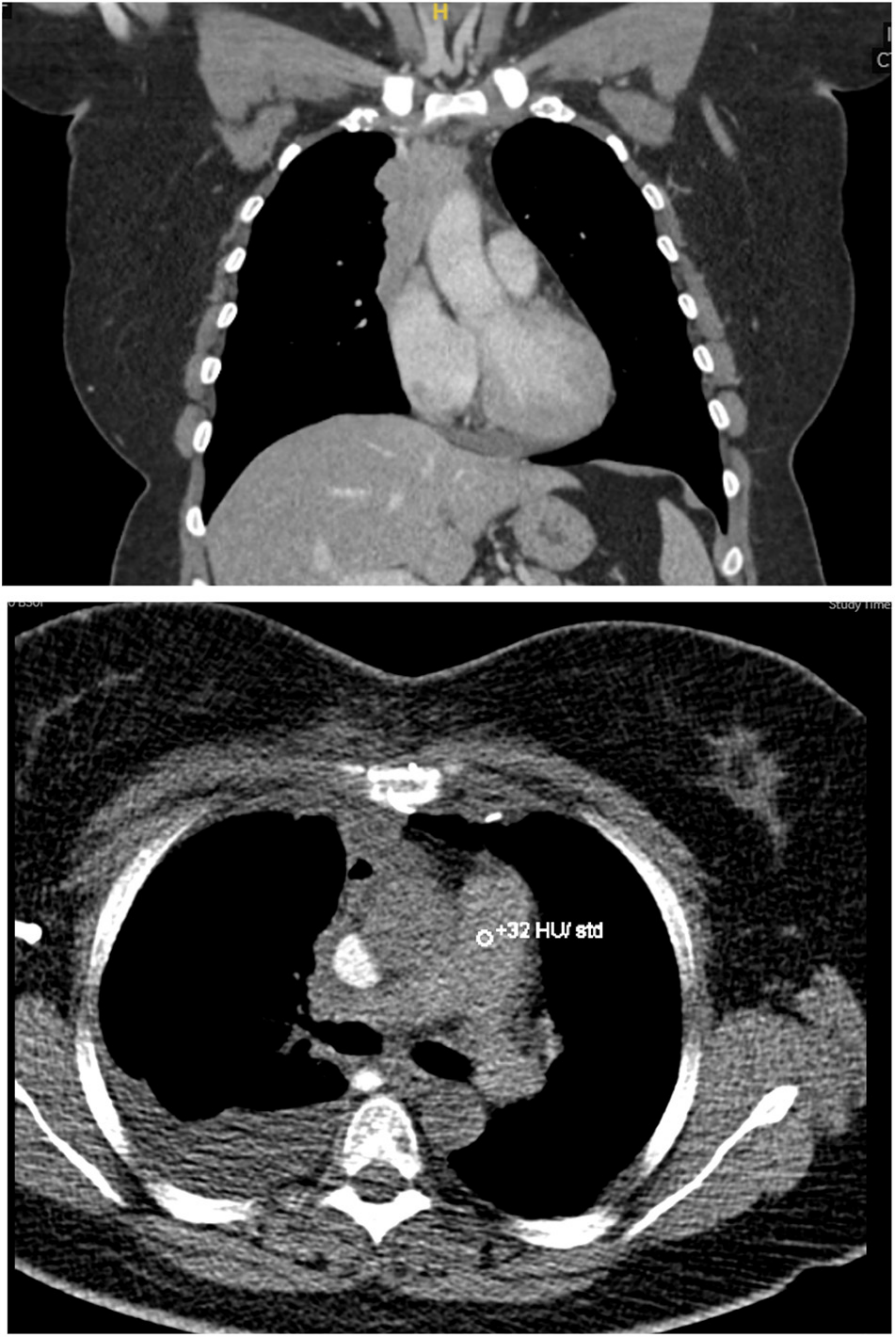
Staged procedure, median sternotomy + radical thymectomy with right innominate vein reconstruction followed by right thoracotomy + extrapleural pnuemonectomy + laser ablation.

**Figure E2:**
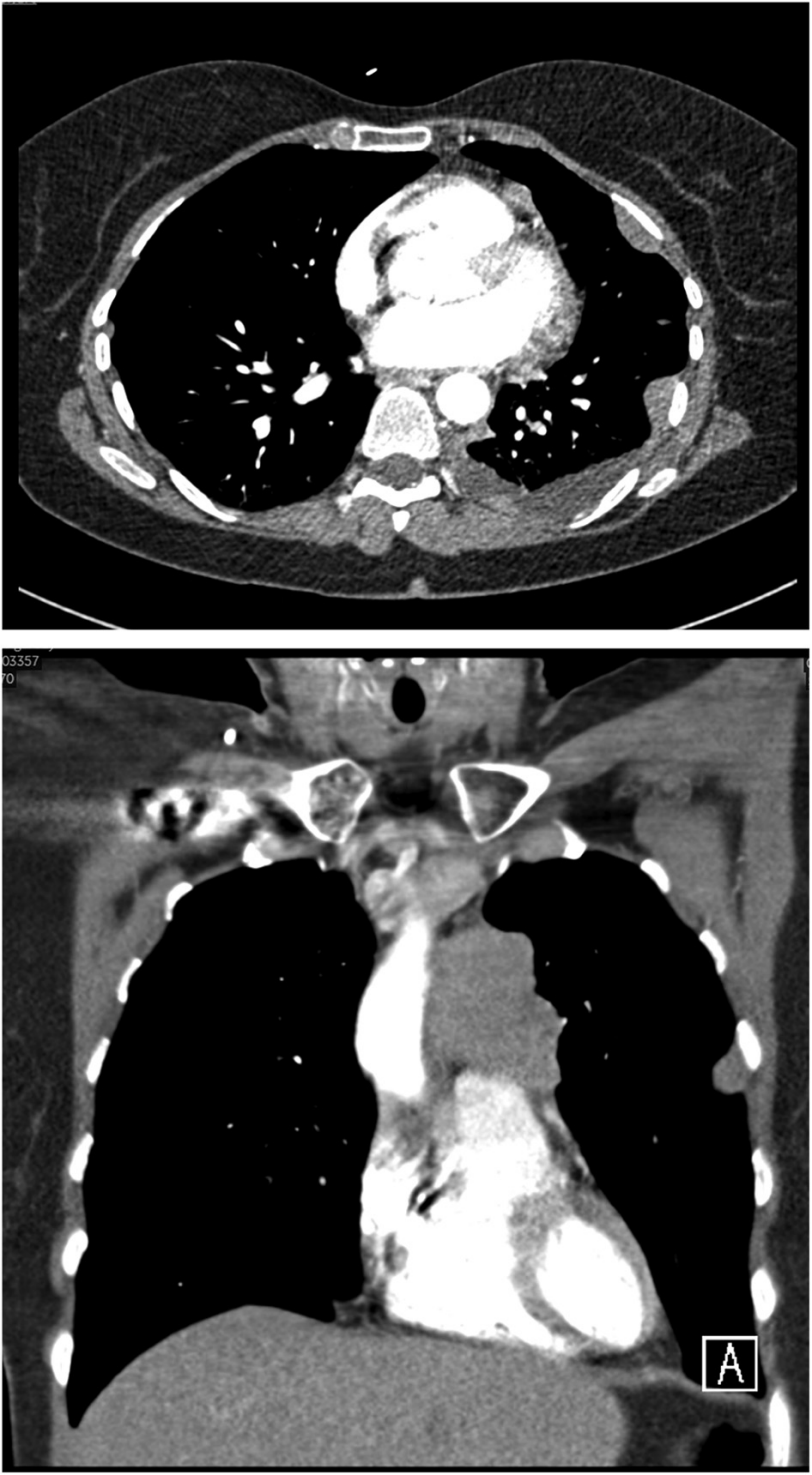
Staged procedure, left thoracotomy + extrapleural pnuemonectomy, followed by median sternotomy + radical thymectomy.

Two patients had planned staged procedures to allow an easier recovery from surgical insult, as per the surgeon's preference. The more complex part was undertaken first in order to confirm resectable disease. One patient underwent left thoracotomy + EPD, followed by median sternotomy + radical thymectomy, at an 8-week interval (Figure E2). One patient had a median sternotomy + radical thymectomy with right innominate vein reconstruction followed by right thoracotomy + EPD + laser ablation, at an 8-week interval (Figure 2). Cardiopulmonary bypass was never required but remained on standby.

One patient had a right EPD for recurrent thymoma. She initially had a right video-assisted thoracic surgery (VATS) thymectomy (2008), followed by a redo right VATS localized pleural resection and resection of the fourth rib for metastatic disease (2019). Two years later (2021), she had local recurrence and subsequently underwent a right EPD + laser ablation for disseminated pleural disease with lung deposits.

Two patients had surgery for recurrence after their initial extended resections. One had local recurrence after right EPD (R1) and 3 years later underwent redo thoracotomy + right chest wall resection/reconstruction. The other patient initially had a left EPD, then developed distant recurrence in the form of a pleural-based lesion in the contralateral lung. She underwent right VATS excision of this pleural-based mass (R1) 1 year after the initial resection.

Both of the patients who underwent EPP procedures had resection of the ipsilateral hemidiaphragm and the pericardium. Three of 8 patients that underwent EPDs had diaphragm resection, 4 of 8 pericardial resection, and 6 of 8 had additional lung parenchyma resected due to invasion. Laser ablation with the Limax 120 Nd:Yag laser (Gebrüder Martin GmbH & Co KG) was used in the latter 3 cases to treat pulmonary and chest wall deposits.

Length of stay ranged from 2 to 21 days, with a median of 7 days. Seven of 10 patients experienced postoperative complications. The most common complication was lower respiratory tract infection (5/10 patients). Two patients were readmitted to hospital within 30 days: one patient for pain management and the other for a myasthenic crisis (Table E1).

There was no intraoperative or in-hospital mortality. All patients were alive at 90 days. Nine of 10 patients were independent with activities of daily living by their first outpatient follow-up clinic appointment (Table E2).

Histologic tumor types were thymoma type AB (20%), B1 (10%), B2 (50%), and B3 (20%) (Table 4). In total, 60% of patients had R1 resection. No patients had R2 resection; however, RX was reported in 3 cases for diffuse tumor spread. All patients were rediscussed at the local lung cancer MDT meeting postoperatively with formal histology available.

**Table 4:** Pathology

Patient	WHO classification of thymic tumors	R status
1	B2	1
2	B1	0
3	B3	1
4	AB	X
5	B2	1
6	B3	X
7	AB	1
8	B2	X
9	B2	1
10	B2	1

*WHO*, World Health Organization.

All patients had adjuvant therapy, the majority receiving adjuvant radiotherapy (8/10). Two patients had chemotherapy recommended by the MDT: 1 because the initial surveillance CT scan demonstrated early recurrence and the other because the patient had already had 2 episodes of recurrence.

Seven patients had disease recurrence with an average disease-free interval of 44 months (range, 8 months to 10 years). Of the 8 patients who have completed 5-year follow-up, survival is 88%. Only 3 patients have completed 10-year follow-up so far (Table 5).

**Table 5:** Oncologic follow-up

Patient	Recurrence	Local recurrence	Distant recurrence	DFI, mo	5-y survival	Overall survival
1	Y	Y, chest wall	N	101	Alive	Dead
2	Y	Y, pleural	N	30	Alive	Dead
3	Y	Y, mediastinal	Y, abdomen	98	Alive	Dead
4	N	N	N	−	Alive	Alive
5	N	N	N	−	Alive	Alive
6	Y	Y, mediastinal	Y, abdomen	13	Dead	Dead
7	Y	Y, mediastinum	N	131	Alive	Alive
8	Y	Y, pleural	N	52	Alive	Alive
9	N	N	N	−	Alive	Alive
10	Y	Y, pleural, chest wall	N	8	Alive	Alive

*DFI*, Disease-free interval; *Y*, yes; *N*, no.

## Discussion

This series, along with others, supports the use of extended resections in selected patients with Masaoka-Koga stage IV thymoma as part of multimodality treatment.^3^^,^^11^, ^12^, ^13^ With no operative mortality and similar outcomes to that reported in the literature, surgery can be considered a safe option that has potential to prolong disease-free survival and overall survival.^4^

Current evidence demonstrates that complete resection for thymoma is the most important factor for preventing recurrence and overall prognosis.^14^ Although the majority of our patients had R1 resection, our recurrence rate over the surveillance period was 70%, similar to that quoted in the literature: 71% (34%-100%). Although not all patients have completed 10 years follow-up, overall mortality is currently 40%, compared with a reported mean of 42% (26%-58%).^4^ Our sample is too small to allow further analysis or subgroup comparison.

The National Comprehensive Cancer Network (NCCN) and the ESMO have similar recommendations for surgery in primary and recurrent thymoma. Although ESMO and NCCN both recommend upfront surgery for advanced thymoma if the tumor is considered resectable, NCCN acknowledges that induction therapy before resection may still be advantageous.^4^^,^^15^ We adopted the NCCN approach in trialing induction therapy to help facilitate complete resection. Unlike in lung cancer, where neoadjuvant treatment may distort tissues planes and cause adhesions, our experience of neoadjuvant therapy in thymoma was that it did not compromise or complicate the resection.

In cases of unresectable stage IV thymoma, the patient is referred for a platinum-based chemotherapy in the first instance.^4^^,^^15^ The role of immunotherapy in thymoma management is still in phase 1-2 trials.^16^ Checkpoint inhibitors may potentiate symptoms in patients with autoimmune disorders.^16^ This may affect approximately one third of all patients with thymoma.^12^^,^^13^

Intraoperative use of photodynamic therapy, chemotherapy lavage, and heated povidone-iodine are experimental techniques in addition to EPDs and EPPs for the management of mesothelioma, targeting the problem of residual microscopic disease.^17^^,^^18^ More recently, laboratory-based studies using povidone-iodine have seen positive outcomes in inducing death of human thymic epithelial tumor cells, and in the future could serve a role in tackling micrometastatic disease as part of multimodality treatment for Masaoka-Koga stage IV thymoma.^17^^,^^18^

### Strengths

The largest contribution to the literature of patients with Masaoka Koga stage IV thymoma undergoing surgery is from the European Society of Thoracic Surgeons Thymic Working Group curating data from 152 patients from 12 institutions, across 8 countries, over a 30-year period. Our study contributes 10 patients, which appears high volume for a single center over a 10-year period.^6^

This case series outlines Masaoka Koga stage IV thymoma treated with surgical procedures typically used for a pleural pathology, mesothelioma. Because our series focuses on thymoma with pleural involvement, zero operative mortality reinforces that these transferable procedures are safe when performed by specialists in a center with experience in treating mesothelioma.

Recent publication of MARS 2 (Extended pleurectomy decortication and chemotherapy vs chemotherapy alone for pleural mesothelioma), which compared outcomes in patients with MPM when randomized to EPD or EPD and chemotherapy, emphasizes the importance of appropriate diagnosis, staging, and patient selection.^19^ Extended resections (EPD or EPP) in our cohort of patients with Masaoka-Koga stage IV thymoma did not result in any perioperative deaths, nor did it prevent our patients from undergoing adjuvant therapy due to poor health status after surgery. American Society of Clinical Oncology and ESMO guidelines now recommend PET-CT to evaluate resectability of MPM (2015), a guidance that changed during the recruitment period for the MARS 2 study.^19^ Similar to the MARS 2 cohort, 3 of 10 of our thymoma patients did not have PET-CT; however, these patients were from the former half of our study. We would recommend PET-CT is used for all patients with pleural thymoma before surgical resection.

Although the choice of neoadjuvant and adjuvant treatment was recommended by the specialist MDT, evidence for adjuvant radiotherapy and chemotherapy in thymoma is variable throughout the literature and is also associated with a number of side effects.^20^ In general, neoadjuvant therapy should reduce the tumor size to help facilitate surgery; and adjuvant therapy is used when resection is incomplete.^4^^,^^15^ In the case of advanced thymomas with diffuse spread and pleural involvement, R0 resection is significantly more challenging to achieve, but also to confirm. Although resection may be done with curative intent, residual microscopic disease is likely.^5^^,^^14^

The use of laser in thoracic surgery has been documented for almost 40 years^21^ and is frequently used as part of routine clinical practice for pulmonary metastasectomy at our unit. In 2 cases the Limax 120 Nd:Yag laser was used to provide an alternative parenchymal-sparing approach to traditional stapled wedge resection for pulmonary deposits.^22^^,^^23^

A precise hand-held device delivers laser therapy to the lesion, cutting through the lung tissue around the deposit while vaporizing and coagulating the remaining lung surface. The laser was also used where localized chest wall disease had been resected, as these areas were thought to be at high risk of recurrence.

### Limitations

Our study is limited by its small numbers, retrospective nature, and reporting from a single institution. Without a control group to serve as a baseline for comparison, the findings may be influenced by selection bias. To date, there are no randomized trials, nor any prospective evidence in the literature, to provide a definitive management strategy, and it seems unlikely that randomized data will ever be produced with a condition this rare.

Guidelines support the use of surgery in advanced thymoma if the tumor is deemed resectable, as complete resection is associated with positive outcomes and is superior to other treatment modalities.^4^^,^^15^ In our study 60% of patients had R1 resection. “Resectability” is defined as complete removal of the tumor. However, whether the tumor is resectable is subjective to the MDT meeting, guided by the surgeons present and their own knowledge, experiences and skills. In tumors with pleural involvement, resectability is not as clear cut due to distribution of disease in combination with operative technique. If radical surgery for thymic tumors with pleural involvement were treated like radical surgery for resectable MPM, the overall aim would be to achieve R1 resection.^19^^,^^24^ R1 resection is considered to be the maximum radicality possible for MPM,^24^ and in our opinion, should apply to all radical pleural resections.

In patients with myasthenia gravis, a thymectomy can be performed for symptomatic relief. In this case series of Masaoka-Koga stage IV thymoma, the primary aim was oncologic, to prolong survival, and it is unknown whether removing tumor bulk reduced the symptomatic burden from myasthenia gravis as this was not explored.^25^^,^^26^

## Conclusions

Extended resections such as EPP and EPD are not part of routine thoracic practice in the United Kingdom. We suggest that these cases are referred to dedicated centers with the required expertise. We also suggest the following as possible criteria for optimal patient selection for extended resections of pleural thymic disease would be: (1) Masaoka-Koga stage IV thymoma with pleural involvement; (2) disease confined to one hemithorax; (3) no distant metastases; and (4) World Health Organization performance status 0-1.

## Conflict of Interest Statement

The authors reported no conflicts of interest.

The *Journal* policy requires editors and reviewers to disclose conflicts of interest and to decline handling or reviewing manuscripts for which they may have a conflict of interest. The editors and reviewers of this article have no conflicts of interest.

## Figures and Tables

**Table E1:** Operative course

Patient	Chest drain duration	Postoperative complications	Length of stay
1	2	LRTI and AF	10
2	4	0	4
3	6	Contralateral pneumothorax requiring chest drain	10
4	3	LRTI	4
5	4	0	4
6	1	LRTI	21
7	1	LRTI and recurrent pneumothorax requiring chest drain insertion. Readmission with pain.	13
8	1	0	2
9	1	Readmission with type 2 respiratory failure secondary to myasthenia gravis	3
10	4	LRTI	10

*LRTI*, Lower respiratory tract infection; *AF*, atrial fibrillation.

**Table E2:** First clinic review

Patient	Pain	Cough	Breathlessness	Wound	Independent with activities of daily living
1	Y, occasional	N	Y, on exertion	Healed	Y
2	N	N	N	Healed	Y
3	Y, frequent	Y	Y, at rest	Healed	N
4	Y, occasional	N	N	Healed	Y
5	Y, occasional	N	Y, on exertion	Healed	Y
6	N	N	N	Healed	Y
7	Y, frequent	N	Y, on exertion	Healed	Y
8	Y, frequent	N	N	Healed	Y
9	Y, occasional	N	N	Healed	Y
10	N	N	Y, at rest	Healed	Y

*Y*, Yes; *N*, no.
